# A Long-Term Study of Sons of Alcoholics

**Published:** 1995

**Authors:** Marc A. Schuckit

**Affiliations:** Marc A. Schuckit, M.D., is a professor of psychiatry at the University of California, San Diego, School of Medicine, and director of the Alcohol Research Center, Veterans Affairs Medical Center, San Diego, California

**Keywords:** children of alcoholics, male, hereditary factors, environmental factors, risk factors, family AODU (alcohol and other drug use) history, AODE (alcohol and other drug effects), predictive factor, behavioral and mental disorder

## Abstract

Men with a family history of alcoholism appear to have a lower intensity reaction to alcohol’s effects than those without this family history. This study investigated whether a lower reaction could encourage greater alcohol consumption among family history-positive (FHP) subjects, predisposing them to develop alcohol-related problems. A family history of alcoholism was associated with increased risk of alcohol dependence and abuse among study subjects. Likewise, over half of the FHP’s whose reactions to alcohol were low had developed alcoholism at a 10-year followup.

The first step leading to the current investigation involving sons of alcoholics was the recognition that alcoholism (usually meaning alcohol dependence^1^) was likely to be genetically influenced. By 1970 it was apparent to researchers that alcoholism runs strongly in families, and studies of twins demonstrated that a higher risk for this disorder is associated with a greater level of genetic closeness to an alcoholic person^2^ (Schuckit 1994*a*). In acknowledgment of this important earlier work, several studies demonstrated that the risk for alcoholism in a person is best predicted by severe alcohol problems in a biological parent, not by the pattern of problems in an adoptive or rearing parent (Goodwin et al. 1973; Schuckit et al. 1972). (For further discussions of twin and adoption studies, see the articles by Cadoret, pp. 195–200, and Prescott and Kendler, pp. 200–205.)

The studies from the early 1970’s also revealed how complex the genetic influences in alcoholism are likely to be. It became apparent that the mode of inheritance of alcoholism does not follow any simple single gene but is more likely to involve multiple genes or a limited amount of genetic material that is not always expressed. The twin and adoption studies also affirmed that genes are unlikely to be the sole cause of alcoholism. Instead, they appear to interact with environmental events to produce a higher or lower level of risk. These investigations also revealed that it is likely that many different ways exist for a person to be genetically at higher or lower risk for alcohol dependence (Schuckit 1994*a*).

## Study Preparations

In 1975, in response to these complexities, preliminary studies were begun to determine whether it was possible to identify some genetic factors that interact with the environment to increase the risk for severe alcohol-related problems over the life span. The plan first called for a comparison of family history-positive (FHP) subjects (i.e., relatives of alcoholics) with family history-negative (FHN) controls. The research group began by hypothesizing that people at higher and lower risk for alcoholism may differ in their personality structure, their cognitive or thinking styles, or their reactions to alcohol. The latter idea grew out of reports from patients that from early on in their drinking careers, they had been able to consume large amounts of alcohol with relatively little effect. As described in other papers (Schuckit 1994*a*), the research group conjectured that a lower intensity of reaction to alcohol might encourage some people to consume greater amounts during most drinking sessions. This pattern, in turn, could make such people more likely to develop alcohol-related life problems. Subsequently, the research group hoped that if it identified differences between FHP’s and FHN’s, particularly with respect to alcohol reaction intensity, it would be able to follow up with the subjects a decade or so later to see whether those earlier differences predicted who developed alcoholism (Schuckit 1994*a*). This article briefly reviews the results of this ongoing investigation.

## The Research Approach

After the preliminary studies helped to improve research methods, a questionnaire was used each year from 1978 to 1988 to identify Caucasian males between ages 18 and 25 who drank but who were not alcohol dependent. Those men who reported problems consistent with alcoholism in their biological fathers were selected as the FHP’s, or high-risk subjects. For each FHP, an FHN (with no family history of alcoholism) was selected based on similarity with respect to age; race; educational level; and pattern of alcohol, tobacco, marijuana, and other drug use. The similarity of drinking patterns was important to ensure that any FHP–FHN differences in the reaction to alcohol were not just a reflection of differences in early drinking practices. To avoid any possible impact of fetal alcohol syndrome, the subjects selected had an alcoholic father but not an alcohol-dependent mother.

All subjects were brought to the laboratory, where face-to-face interviews were conducted to verify the subjects’ family histories and drinking status, and personality and cognitive tests were administered. All subjects then received a dose of alcohol ranging from approximately three to five drinks (0.75 to 1.1 mL of ethanol per kg of body weight). In an additional session, all but the earliest group of subjects (because the study had not yet been refined) received a placebo, believing that they had been given alcohol. After consuming the beverages, the subjects’ biological and perceptual changes were evaluated over the next 3 hours. These postdrink measures, recorded every 15 to 30 minutes, included evaluations of the subjects’ own feelings of intoxication; evaluations of the more biological changes associated with drinking (such as alcohol-related alterations in hormones and brain waves); and changes in motor performance, using body sway as an indicator (Schuckit and Gold 1988; Schuckit 1994*a*).

Between 1978 and 1988, the reactions to alcohol were evaluated for 453 men. Subsequently, between 1989 and 1994, when the subjects were about 30 years old, steps were taken to locate them. The goal was primarily to determine the relationship between the subjects’ intensity of reaction to alcohol (defined by the combination of their subjective feelings and biological changes following an alcohol challenge) and their future risk for alcohol-related problems (Schuckit 1994*a*,*b*; Schuckit et al. 1994). The role in this relationship of the subjects’ family histories of alcoholism, as well as the role of psychiatric disorders, was investigated. The followup evaluations were carried out by researchers who knew nothing about the subjects’ statuses at age 20. To corroborate the information provided by the subjects, an interview was carried out with an additional informant (usually the spouse), and blood tests (indicators of whether heavy drinking had recently occurred) and urine samples for drug toxicology screens were obtained (Schuckit 1994*b*).

## Findings

### Comparing Populations

Despite a clear family history of alcoholism in one group and no family history of severe alcohol problems in the other, the FHP’s and FHN’s demonstrated no consistent differences on personality measures (Schuckit et al. 1994). Nor were any differences observed on cognitive test results in the group of FHP’s and FHN’s matched with respect to educational level (Schuckit 1994*a*; Schuckit et al. 1987*a;* Schuckit et al. 1990). Following the alcohol challenges, the FHP’s and the control group also had virtually identical blood alcohol concentrations during the 3-hour testing session (Schuckit and Gold 1988; Schuckit 1994*a*). This result was consistent with the manner in which the two family history groups had been carefully matched in their usual drinking patterns and drug-use histories.

**Figure 1:**
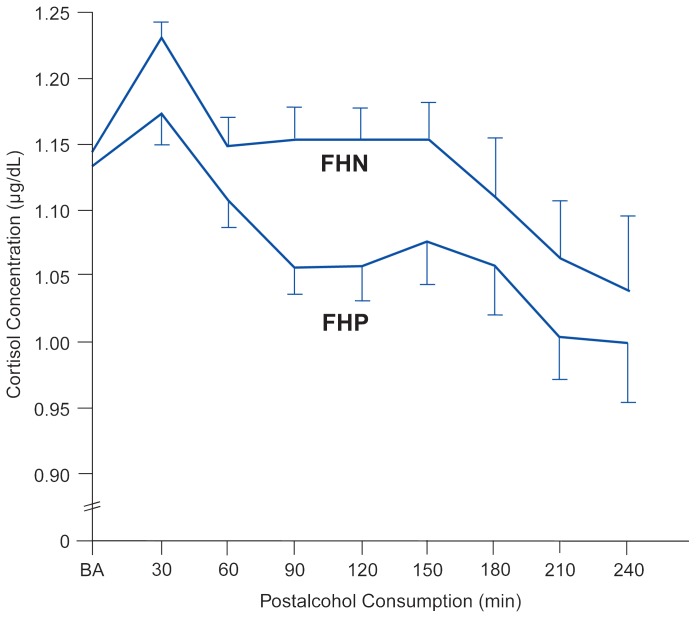
The hormone cortisol was measured in both family history-positive (FHP) and family history-negative (FHN) subjects before consuming alcohol (i.e., at baseline [BA]) and at points during the 3 hours after receiving 1.1 mL/kg of alcohol (equal to four or five drinks). As shown here, FHP subjects experienced a lesser change in cortisol levels than did FHN subjects, demonstrating the FHPs’ lower levels of response to alcohol.

Despite the similarities between FHP’s and FHN’s, approximately 40 percent of the sons of alcoholics but fewer than 10 percent of the control subjects demonstrated remarkably low levels of reaction to the alcohol they were given (Schuckit and Gold 1988). Figure 1 depicts one example of this finding using a biological measure—the changes in the hormone cortisol following the alcohol-consumption challenge (Schuckit et al. 1987*b*). Cortisol level, however, is only one measure. Similar types of FHP–FHN differences, each demonstrating a lower level of response to alcohol among the sons of alcoholics, were shown for subjective feelings of intoxication, levels of standing steadiness (i.e., body sway), two additional hormones, and two different measures of brain activity (Schuckit 1994*a*).

### Followup

Having demonstrated a lower level of response to alcohol among the sons of alcoholics across different alcohol doses and using different measures over the years, the next step involved the followup. As described in more detail in several recent publications (Schuckit 1994*a*,*b*; Schuckit and Smith in press), all 453 men were successfully located an average of 8.3 years following their initial testing. Almost all these subjects (i.e., 450 of 453 men, or 99.3 percent) participated in the followup evaluation, which involved a face-to-face interview with each subject and with an additional informant, such as a spouse. As reported in a recent manuscript, focusing on the men for whom a family history could be clearly established (e.g., after excluding FHN subjects for whom alcohol problems developed in a more distant relative during the followup), a family history of alcoholism was associated with an almost threefold increased risk for alcohol dependence and at least a doubling of the rate for alcohol abuse (Schuckit and Smith in press). At the same time, the FHP’s had no marked increased risk for abuse of or dependence on marijuana-type drugs (i.e., cannabinols), although they showed a trend for increased risk for stimulant abuse or dependence. Sons of alcoholics had no higher-than-expected rates of psychiatric disorders (except for alcoholism). The latter finding argues against the possibility that the subjects who went on to develop alcoholism might have been self-medicating a psychiatric problem.

**Figure 2:**
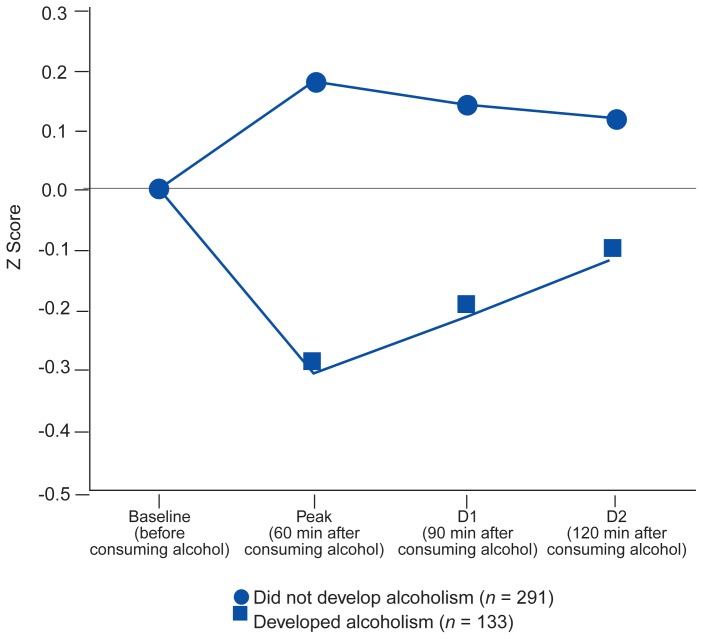
Responses to alcohol (Z scores) were compared for 424 subjects who were followed for more than 8 years to monitor whether they developed alcoholism. Z scores show how far a subject’s reaction to alcohol differs from the average response. Levels of reactions were based on subjective reports and changes in body sway and the hormone cortisol. Those who later developed alcoholism had much lower Z scores at their peak blood alcohol concentrations and at subsequent time points, D1 and D2, than those who did not develop alcoholism.^1^ ^1^Only subjects for whom all relevant data were available were included.

The results of the major focus of the study—the relationship between the level of response to alcohol early in life and the future risk for alcoholism—were fairly clear. Analysis of the data from the first 223 subjects revealed that 56 percent of the FHP’s with lower levels of alcohol response developed alcoholism during the period before followup, compared with only 14 percent of those with high levels of sensitivity to alcohol (Schuckit 1994*b*). Figure 2 presents data from the sample of more than 400 men who could be clearly classified regarding family history, comparing the reactions to alcohol at about age 20 for those men who later developed alcoholism with the reactions for the men who did not. The data reveal a much lower response to alcohol for the men who later developed alcohol abuse or dependence. It appears that at least among the subjects who had very high or very low levels of response to alcohol early in life, much of the family history’s ability to predict future alcoholism appeared to operate through this level of response to alcohol (Schuckit and Smith in press).

## Putting These Findings in Perspective

This long-term study of Caucasian men at high and low risk for alcoholism suggests several conclusions. First, the data reaffirm how important family history is in predicting future alcoholism. Second, the level of response to alcohol at approximately age 20 is both significantly lower among men at high risk for developing this disorder (i.e., sons of alcoholics) and appears, by itself, to be a fairly potent predictor of future alcoholism risk. Thus, both FHP’s and FHN’s with the lower level of response to alcohol had high rates of subsequent alcohol abuse or dependence. Third, these data do not indicate that (at least for this sample of 450 relatively functional young men) the future risk for alcoholism is related to any higher risk for the development of a major psychiatric disorder (such as severe depressive episodes or severe anxiety) before the onset of alcoholism. Finally, the most recent analyses indicate that for those individuals with extreme levels (i.e., very high or very low) of response to alcohol, much of the family history’s ability to predict the future risk for alcoholism relates to the low level of response to alcohol.

### New Research Directions

At the same time, these study results demonstrate how complex the risk for alcoholism seems to be. For example, some men who showed the low response did not develop severe problems with alcohol. Thus, for the 15-year followup of these same subjects, the research group is gathering information about environmental and psychological factors that may interact with the level of response to alcohol to help protect people who are at risk for alcohol abuse or dependence. Some of the additional factors being studied include levels of life stress, ways of coping with stress, and drinking and drug use by peers. Identifying protective environmental, interpersonal, and other factors that interact with biological influences is a major goal of the current work.

This ongoing stage of followup also will allow the research group to observe an important population of women as well as men. Additional information is therefore being gathered about the sons and daughters of the original subjects (i.e., the grandchildren of alcoholics).

Of course, the studies described here evaluate only one part of the picture. Some men and women develop alcohol problems while in their teens, often in the context of severe and repeated antisocial problems. Such subjects were not included in this investigation. Other alcohol-dependent people may have developed their disorder in association with schizophrenia or another severe psychiatric disorder; these people were not included in this work if they had experienced an early onset of their psychiatric problem. The finding that the majority of alcoholics do not have severe psychiatric disorders, however, is consistent with additional studies from this research group (although alcoholics do demonstrate temporary psychiatric problems in the context of their intoxication or withdrawal) (Schuckit 1994*a*).

The studies described here also are limited to Caucasian and moderately functional subjects who had jobs or were in school when originally selected. To determine if similar results could be seen in another group of people, the findings recently have been replicated in a totally different sample of children of alcoholics selected as part of the research for the Collaborative Study on the Genetics of Alcoholism (Schuckit et al. in press). This group is more racially diverse and includes male and female subjects from all parts of the country.

The potential importance of a low level of response to alcohol has been corroborated by a recent overview, or meta-analysis, of studies comparing FHP’s and FHN’s (Pollock 1992). At the same time, other studies—often using different alcohol doses, different modes of administering alcohol over an extended period of hours, or additional challenges during the test session (such as electric shock)—do not always show the same results as those reported here (Newlin and Thomson 1991; Bauer and Hesselbrock 1993). Impressive work from other laboratories also indicates that additional biological factors, such as brain waves, might be associated with enhancing future alcoholism risk (Begleiter and Porjesz 1988).

## Summary

This article has reported data from a series of studies that span the period from 1970 to 1995. These investigations support the importance of genetic influences in alcoholism but emphasize that subgroups of alcoholics exist whose disorder reflects different genetic and environmental factors. One of many potential characteristics that might increase the probability that a person will drink more heavily and more often is a lower level of intensity of reaction to alcohol. Some people may therefore develop alcohol-related problems because they seek a response to alcohol that they can only perceive at higher levels of alcohol intake, a pattern that in itself increases the probability of the need for ever-greater amounts of alcohol and subsequent alcohol-related problems.

## Footnotes

1 As it is used in this article, the term “alcoholism” refers to the definition of alcohol dependence stated in the American Psychiatric Association’s *Diagnostic and Statistical Manual of Mental Disorders, Fourth Edition*.

2 Identical twins have the same genetic makeup. Therefore, they possess greater genetic closeness than do fraternal twins.
